# Planting period is the main factor for controlling maize rough dwarf disease

**DOI:** 10.1038/s41598-020-79994-5

**Published:** 2021-01-13

**Authors:** Gemma Clemente-Orta, Ramon Albajes, Iván Batuecas, M. A. Achon

**Affiliations:** 1 Department of Crop and Forest Sciences, AGROTECNIO Center, University of Lleida, Rovira Roure 191, 25198 Lleida, Spain; 2 Departament de Protecció Vegetal, IRTA-Centre de Cabrils, 08348 Cabrils, Spain

**Keywords:** Environmental sciences, Ecology, Agroecology, Behavioural ecology, Community ecology, Ecological epidemiology, Ecological modelling, Ecological networks, Ecosystem ecology, Ecosystem services

## Abstract

Maize rough dwarf virus (MRDV) is one of the main yield-limiting factors of maize in the Mediterranean. However, knowledge about the interactions between the agroecosystem and the virus–vector–host relationship continues to be limited. We used multi-model inference to test a landscape-scale approach together with variables measured in the field, and we estimated the effects of early and late planting on MRDV incidence. The results revealed that the virus incidence increased by 3% when the planting was delayed, and this increase was coincident with the first peak of the vector population. The variables at the field and landscape scales with a strong effect on virus incidence were the proportions of grasses in adjacent crops, in uncultivated areas, and in edges close to maize plants. Grass plant cover in the edges also affected virus incidence, but these effects varied with the planting period. These findings provide new insights into the causes of MRDV incidence and may provide some guidance to growers to reduce losses caused by the virus. Among the recommendations to be prioritized are early planting, management of grasses at field edges, and non-overlapping cultivation of maize and winter cereals in the same area.

## Introduction

Global agriculture is evolving in response to human population growth, a growing demand for different food commodities, climate change, and new issues related to agriculture, such as biofuels, the agro-pharma industry and CO_2_ absorption. Thus, global agriculture can be the single largest driver of global environmental change if it combines sustainable practices and meets human needs^1^. Viruses are the second most important group of plant pathogens that cause substantial losses, mainly in intensively cultivated crops^2,3^. While the management of agricultural habitats offers solutions to reduce yield loss due to pests^4^, in the case of viral diseases, the oversimplification of crops and genetic cultivar diversity, intensive farming systems and the increasing use of phytosanitary products have interfered with the ecological functions of agroecosystems and have altered the epidemiology of plant diseases^5^.

The host plant, vector and virus are interdependent components of a complex pathosystem. Thus, it has been suggested that the spread of infectious diseases is inherently a spatial process often embedded in physically complex landscapes^6^. However, few studies have addressed the link between spatial processes at the landscape scale, the ecology of vectors in crop colonization and virus transmission in the disease epidemiological process^7^.

Maize rough dwarf disease (MRDD) is one of the most damaging viral diseases found in the maize growing areas of Europe, Asia and South America. Spain, other areas of the Mediterranean Basin and Asia are affected by maize rough dwarf virus (MRDV), a member of group 2 of *Fijivirus* (Fam. Reoviridae)^8–13^. In Spain, the occurrence of MRDV was first reported in the 1960s^14^, and a later outbreak of this virus was observed in 1999 in the northeastern region of the country^15^. Intensive surveys conducted from 2001 to 2006 in the main maize growing regions of Spain revealed that MRDV was the most widespread virus infecting maize crops, with an estimated coverage of 68% of the Spanish maize surface area^16^. MRDV is transmitted in a persistent propagative manner by the planthopper *Laodelphax striatellus* Fallén (Delphacidae, Fulgoroidea), which is a unique natural vector for MRDV in Spain that contributes to an increase in viral inoculum^17–19^. Overwintering nymphs carrying MRDV survive in weed grasses, and then, the adults move into and infect maize when feeding on the plants^20–22^. Early infections lead to severe plant stunting and premature death when maize plants are most susceptible^18,23^. Maize is the most widely affected crop in Spain^16^, and the MRDV host range is limited to Gramineae, with lower proportions in species such as *Digitaria sanguinalis* (L.) Scop, *Echinochloa crus-galli* (L.) P.B., *Cynodon dactylon* (L.)^14,17,20^, and *Lolium perenne* (L.); however, this virus is occasionally found in wheat crops, exhibiting a low occurrence rate^18^. Although winter cereals have been shown to act as winter reservoirs for other fijiviruses^24–26^, the role of these crops in MRDV epidemiology in Spain has not yet been defined^16,18^.

It is widely accepted that the epidemiology of MRDD is strictly linked to the abundance and distribution of its vector^17^. In Spain, the population dynamics of *L. striatellus* on maize show abrupt seasonal fluctuations, with one peak in June and another in September^17,18^. These studies determined that the incidence of MRDV was correlated with the first captures in maize fields during the first developmental stages of the crop. However, several additional factors must be analysed to optimize management strategies. In this sense, the planting period also varies among maize growing areas^27–29^ according to the climate conditions, and the expected length of the growing season in areas where maize is produced and the optimal period for planting vary greatly^30^. In the irrigated area of Spain, the maize growers usually sowed maize from March to April; however, in recent years, growers have delayed the planting period, likely as a result of milder springs and earlier winter cereal harvesting allowing the sowing of maize after the winter cereal harvest^31,32^.

This study aimed to identify the landscape and field factors that are mainly involved in MRDD epidemiology in our area. To further extend the knowledge of infection risk drivers, we asked the following questions: (1) can we elucidate, from a landscape perspective, the main epidemiological factors driving the incidence of an endemic virus? (2) What are the landscape and field variables involved in the risk of maize infection by MRDV? (3) Are the same factors involved in maize fields sown early and late?

## Results

### MRDV incidence in maize fields

A total of 1324 maize plants were analysed during two consecutive years. The average virus incidence registered was 12.90% in 2016 and 9.45% in 2017, with no significant differences between years (*F*_1,45_ = 1.04, *p* = 0.3) (see Supplementary Table S1). However, we found significant differences between sowing months (*F*_3,39_ = 4.25, *p* = 0.011). Specifically, the average incidence was higher in the fields sown in May (13%) and lower in those sown in March (1.3%). These results helped to set the two planting periods used in the next analysis.

### Effects of field and landscape variables on MRDV incidence

**Figure 1:**
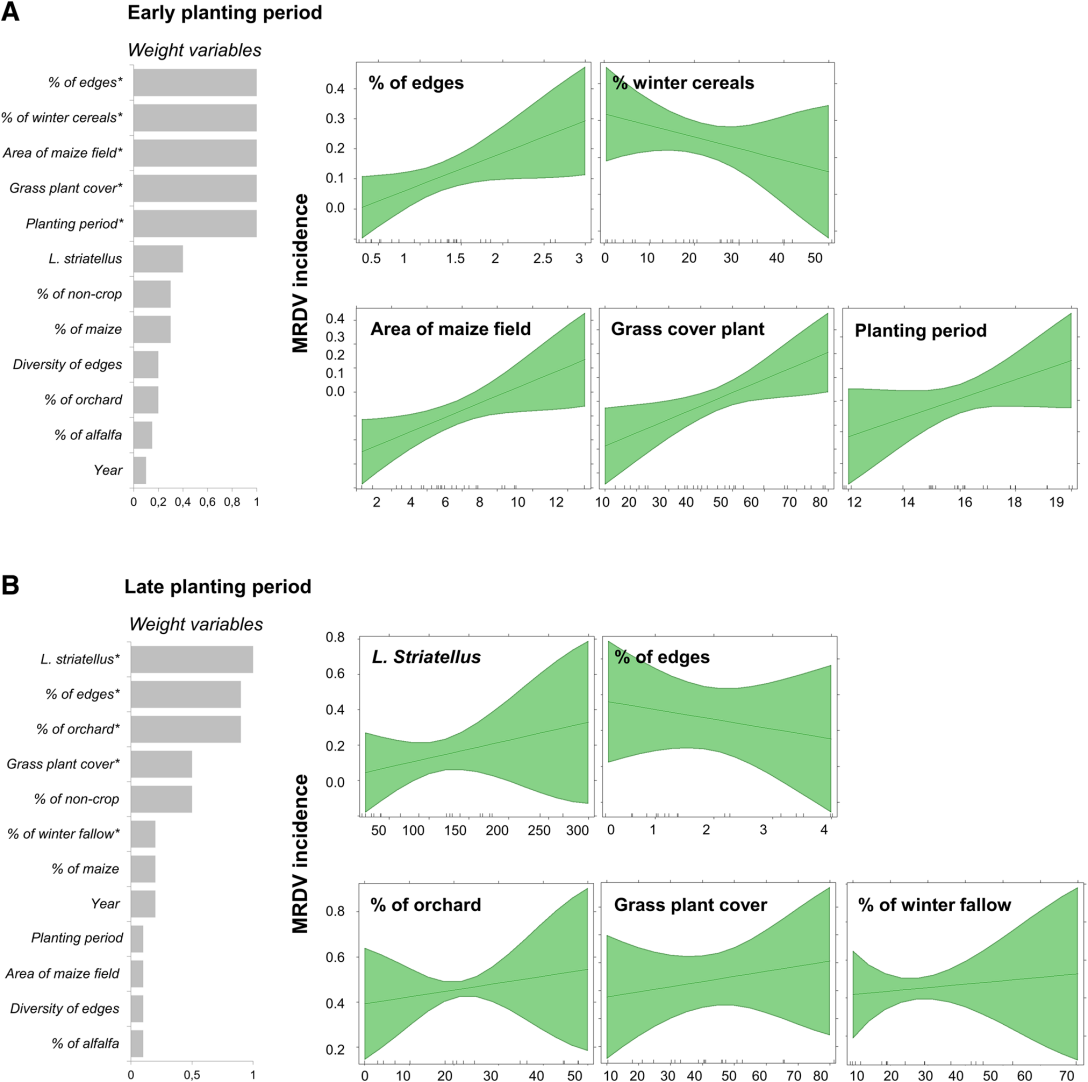
Effects of field and landscape variables on MRDV incidence on early and late planting periods. Generalized linear models (GLMs) using a binomial distribution were used in the model average and multi-model inference to select the best predictor model for MRDV incidence. The grey bars show the relative importance of each predictor variable (i.e., the sum of Akaike weights of the models in which each variable is optimized following the criterion ΔAIC < 2). The green graphs show the effects of significant variables (*) on MRDV models and how they varied depending on the planting period represented by the confidence intervals that contain a wide range of MRDV incidences. Therefore, the differences in incidence caused the interval widths to vary, and this effect was stronger in the late planting period.

The most parsimonious models for MRDV incidence were obtained using a model averaging and multi-model inference approach. The result of the early planting model is shown in Fig. 1A, and that of late planting is shown in Fig. 1B. In grey bars, we show the relative importance of each predictor variable (sum of Akaike weights of the models in which each variable appears in the best models ΔAIC < 2). In the green graphs, we present the effects of significant variables (*) on MRDV models and how they varied depending on the planting period represented by the confidence intervals, which contain a wide range of incidences of MRDV. Different incidence values registered in maize fields caused the interval widths to vary, and this effect was stronger in the late planting period. The most significant variables affecting MRDV incidence varied mainly in the two planting periods analysed. In both planting periods, the grass cover at the edges was positively related to virus incidence, but the effect of other significant variables varied. For the early planting period, the surface area of maize fields, the proportion of edges, and the planting period (number of weeks in the year) were positively related to MRDV incidence, while the proportion of winter cereals in the landscape was negatively related. For the late planting period, the vector abundance and the proportions of both orchards and fallow winter cereal in the landscape were the variables positively related to MRDV incidence. In contrast with the result for the early planting period, the proportion of edges in the late period was negatively related to virus incidence.

### Influence of the phenology of *L. striatellus* flights on MRDV incidence

**Figure 2:**
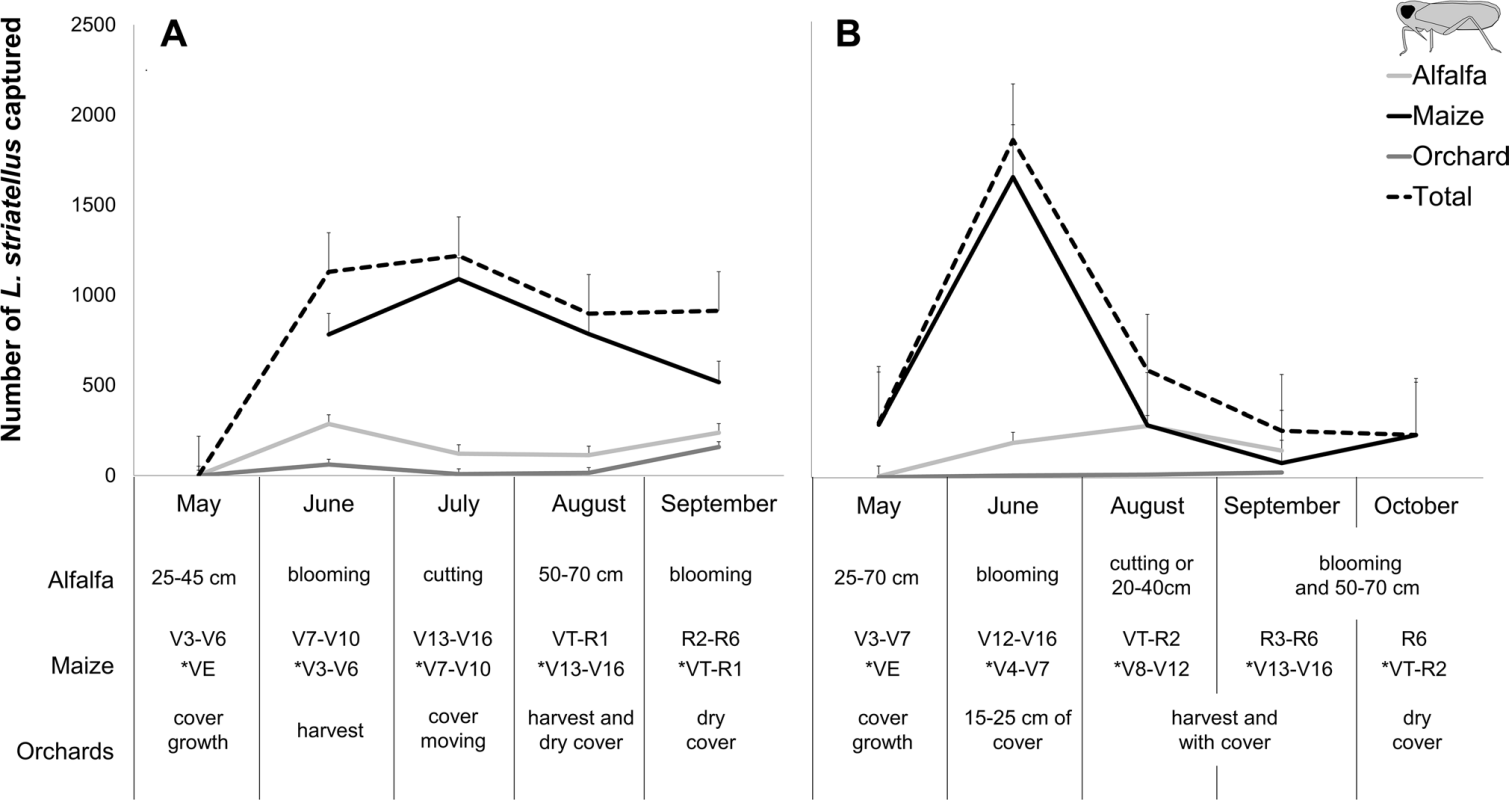
Abundance of *L. striatellus* during the maize growing season. The table shows differences in the phenology and management of sampled crops.

The population dynamics of the MRDV vector monitored with yellow sticky traps (from March 2016 and May 2017) are shown in Fig. 2. A total of 7,451 *L. striatellus* individuals were caught during 2016 and 2017 (4,223 and 3,228, respectively). Furthermore, we found differences between the months in which the vector populations were caught in both 2016 (*X*^2^ = 218.4, df = 5, *p* < 0.001) and 2017 (*X*^2^ = 95.83, df = 4, *p* < 0.001). In 2016, the vectors were more abundant from June to September than in March and May (Dunn test, *p* < 0.001), and in 2017, the vectors were more abundant in June and October than in May, August and September (Dunn test, *p* < 0.001).

Moreover, more *L. striatellus* individuals were captured in maize fields than in alfalfa fields or orchards. The models relating MRDV incidence and the number of vectors trapped showed that the incidence was positively related to vector catches in maize fields in May, with catches in alfalfa fields, maize fields and orchards in June, and with catches in orchards and maize fields in July (Table 1).

**Table 1:** Effects of the phenology of *L. striatellus* flights on MRDV incidence.

	RA (%)	Estimate	z value	*p* value
**May**				
(Intercept)		− 2.06	− 8.56	< 0.001
Alfalfa	4.07	− 0.02	− 0.21	0.83
Orchard	1.36	0.08	0.79	0.43
Maize	94.57	0.27	3.63	**< 0.001**
**June**				
(Intercept)		− 2.12	− 9.31	< 0.001
Alfalfa	15.72	0.20	2.88	**< 0.001**
Orchard	2.36	0.17	2.27	**0.02**
Maize	81.92	0.41	4.69	**< 0.001**
**July**				
(Intercept)		− 2.11	− 23.57	< 0.001
Alfalfa	89.93	− 0.10	− 1.14	0.26
Orchard	5.15	0.41	5.90	**< 0.001**
Maize	4.92	0.32	4.82	**< 0.001**

Vector capture was performed in alfalfa fields, maize fields and orchards during the maize growing season. The models relating MRDV to the abundance of *L. striatellus* captured in crops in sampling months were analysed using a generalized linear mixed model (GLMM) for binomial distribution.

### Margin-covering species and grasses at edges as virus reservoirs

To determine and characterize the cover weed species, a total of 203 plant species were identified in the 504 sampling points during the edge surveys in both years. A total of 64 grass samples identified as *Avena sterilis* L., *Avena sp.* (fallow), *Brachypodium phoenicoides* (L.), *Bromus diandrus* Roth, *Bromus catharticus* Vahl., *Cynodon dactylon* (L.) Pers., *Dactylis glomerata* L., *Echinochloa crus-galli, Eragrostis* spp., *Hordeum murinum* L., *Lepturus* spp., *Lolium rigidum* Gaudin*, Oryzopsis miliacea* (L.), *Phalaris minor* Retz., *Phleum paniculatum* Huds., *Poa pratensis* L., and *Polygonum* spp. were collected and analysed (Table 2).

**Table 2:** Types of edges sampled using the Braun-Blanquet scale.

Type of edge	% of soil covered	Shannon Index	Grasses more abundant ≥ 20%	Species analysed	No. samples analysed
Ground cover of orchards	48.43	1.57	*H. murinum*	*A. sterilis*	2
			*P. annua*	*B. diandrus*	2
			*C. dactylon*	*B.phoenicoides*	1
				*E. crus-galli*	1
				*P. paniculatum*	1
Maize-Alfalfa	48.97	1.70	*S. halepense*	Avena sativa	9
			*A. sterilis*	*A. sterilis*	3
			*B. diandrus*	*B. diandrus*	1
			*H. murinum*	Lepturus sp.	1
			*C. dactylon*	*L.rigidum*	1
			*L.rigidum*	*B.catharticus*	1
			*P. annua*	Polygonum sp.	1
			*B.phoenicoides*	*O. miliacea*	1
				Eragrostis sp.	1
Maize-Maize	70.11	1.66	*S. halepense*	*P. pratensis*	1
			*C. dactylon*	Avena (ricio)	2
				*A. sterilis*	2
				*P. minor*	2
Alfalfa-Orchards	77.38	1.66	*H. murinum*	*A. sterilis*	5
			*C. dactylon*	*B. diandrus*	2
			*B. diandrus*	*B.phoenicoides*	1
			*S.halepense*	*H. murinum*	1
				*B.catharticus*	1
Maize-Orchards	80.00	1.69	*H. murinum*	*A. sterilis*	8
			*S. halepense*	*B.catharticus*	4
			*C. dactylon*	*B.phoenicoides*	1
			*P.annua*	*L.rigidum*	3
				*P.pratensis*	1
Non-crop habitats	91.28	1.92	*D. glomerata	*O. miliacea*	1
			*H. murinum	*A. sterilis*	2
			*A.sterilis	*D. glomerata*	1
				Total	64

*Cover < 15%.

Composition of plant species, cover abundance and the H’ according to the field edges of maize-neighbouring crops. The table shows the most abundant grass (≥ 20% of plant cover) and species analysed by selective isolation of dsRNA from each type of edge.

In Table 2, we show the values for the plant cover and diversity of sampled edges. Overall, the edges of non-crop habitats showed the highest plant cover (91.3%) and H’ value (1.92), while the H’ value of the remaining edges did not differ significantly. The orchard ground cover showed the lowest coverage and diversity (48.4% and 1.57, respectively). In addition, edges between perennial crops and cereals showed a high cover (80% in cereal-orchard and 77.3% in orchard-alfalfa).

None of these samples exhibited MRDV-like symptoms, and no genomic segments of the virus were detected.

## Discussion

Since 1999, MRDD has been the most severe limiting factor for maize production in Spain. Attempts to understand the main factors involved in disease outbreaks have revealed that the introduction of new crop practices and MRDV populations are involved in these disease outbreaks^13,17,18^. Although previous studies have modified cultural practices to reduce MRDD incidence, several questions remain unanswered, probably because the studies were conducted at the field scale and not the landscape scale. Given that the spread of infectious diseases is inherently a spatial process embedded in physically complex landscapes^6^, this study was conducted by taking into account the composition of the landscape surrounding the maize fields in a 500-m buffer area and considering the maize field characteristics as variables involved in the epidemiology of MRDD.

The effects of planting period on virus incidence have been reported by Achon et al*.*^17,18^ for MRDV and by Wang et al*.*^26^ for other fijiviruses. To identify criteria to choose an optimal planting period in our area as a function of the epidemiology of MRDV, we separated the analysis into two periods according to the planting periods of the maize fields sampled. In this manner, we expected to obtain more information about ecological processes involved in MRDV epidemiology, an approach recommended by Chaplin-Kramer et al.^33^ for landscape studies. The choice of these two periods is also meaningful from the perspective of MRDV epidemiology, given that the changing planting periods and the coincidence of the first peak of the vector population were already known in the area. The differences reported in this study showed a 3% reduction in virus incidence in early planting compared to that in late planting, representing an important production benefit for growers.

**Figure 3:**
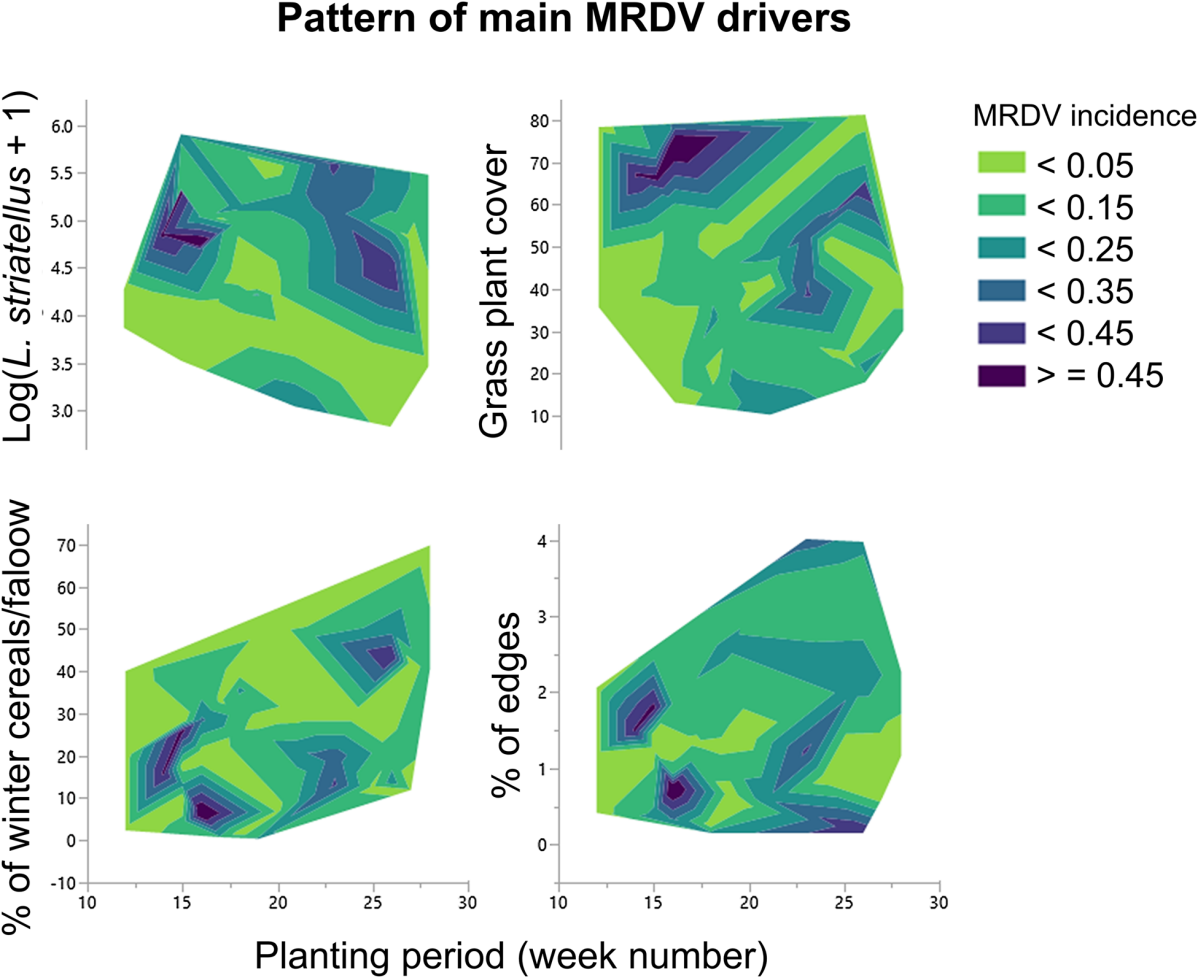
Pattern of the main MRDV drivers. The contour plot contains the following elements: predictors on the X axis (planting period) and Y axis (abundance of *L. striatellus*, proportion of winter cereal/fallow, proportion of edges in the landscape and percentage of grass cover plants at the edges). Contour lines connect points that have the same adjusted response value.

Overall, our findings show an effect of field and landscape variables on virus incidence in the two planting periods (Fig. 3). Specifically, the models showed strong effects of the proportion of edges in the landscape and the grass plant cover in the edges close to maize fields. The fact that MRDV incidence was positively related to these variables while the proportion of winter cereals was negatively related to the variables in early planting confirms the role of grasses in the epidemiology of the virus. This fijivirus is transmitted persistently by a vector that does not thrive or readily acquire the virus when feeding on maize, which is not a breeding host^34^. Therefore, once the vector has acquired the virus while feeding on grasses, MRDV is spread by the vector to the crop and its weeds, particularly when the field is close to grassy patches ^26,35^ in cultivated or uncultivated areas^36,37^.

Thus, winter cereal fields in the matrix of the landscape attract vector adults before spring, as suggested by Achon et al.^17^. Indeed, these areas containing grasses are a feeding and breeding resource for overwintering adult vectors^19,38^. For example, in Italy, perennial host plants of MRDV have not been found, and the virus persists between growing seasons in planthoppers that overwinter as nymphs in diapause^35^. Then, the vector matures and disperses in spring, when the virus is introduced into annual grasses and maize^36^.

Moreover, as the season progresses and winter cereals mature and finally are harvested, the number of vectors that leave winter cereals to colonize nearby young maize plants and grass patches increases. In our area, cereal harvesting from May onwards coincides with the first peak of vector flight by June-July. This can explain the positive relationship between the higher MRDV incidence registered in fields planted later and the proportion of fallow land resulting from cereals harvested in the landscape. Although Achon et al.^17^ had already reported this phenomenon, these authors also remarked that winter cereals are only occasionally a source of MRDV for late-planted maize^18^. The high dispersal capacity of *L. striatellus*^39^ and the propagative type of virus transmission likely allow the vector to retain the infection capacity for longer than non-propagative viruses can. In the case of non-propagative maize viruses such as maize dwarf mosaic virus (MDMV), the closeness of maize field edges also had a strong effect, measured on a spatial scale of 200 m in the maize fields sampled^40^. Moreover, in the early season, grasses could provide the resources needed by the vector for overwintering, while in the late season, grasses in the ground cover of irrigated orchards could remain greener than those at the edges and could be a suitable host for vector reproduction in this area, as remarked by Clemente-Orta et al*.*^41^. Specifically, the ground cover in the orchard was characterized by a higher level of *H. murinum* and *C. dactylon,* and these species could act as potential virus and vector resources^20^. Then, later in the season, when summer is approaching, the young maize plants are more attractive to the vector, and the role of edges as an alternative reservoir is irrelevant, particularly when many edges in the landscape are dry, burned or treated with herbicides by growers. It is important to note that the abundance of winter crops and weed grasses has been reported to be associated with the seasonal abundance of *L. striatellus* at a given site^38,42,43^, whereas the composition of winter grass changes with the season.

On the other hand, among field variables, the maize field surface area and planting period (number of weeks in the year) for the early planting period were variables influencing MRDV incidence. Insect preference for larger fields is a phenomenon that may have several causes; however, during host plant habitat colonization by herbivorous insects, the amount of resources for feeding and reproducing is a major factor^44–46^ affecting habitat selection. Later, when an initial population is present in maize fields, no secondary MRDV infections occur, the infected plants are randomly distributed^47^, and field size is not significantly related to virus incidence in fields planted late.

The population patterns of *L. striatellus* flights on maize were similar in the two study years and affected the seasonal occurrence of this insect reported previously in Spain^17,18^. In addition, these authors reported that the variation in virus incidence was mostly a function of a few viruliferous insects that are required during the early developmental stages of a crop. These results suggest that a higher virus incidence was registered in fields sown later and corresponded mostly to vector migration for the colonization of maize in comparison with the low number of insects caught in March, April and May. As expected, the abundance of vectors in alfalfa fields or orchards was much lower than that in maize. However, despite the low number of vectors in orchards, the virus incidence in the later planting period was positively related to insect catches in orchards and in maize in that period. Clemente-Orta et al*.*^41^ reported that the abundance of *L. striatellus* was related to the proportion of orchards in the landscape in the late season. It is known that different crop management techniques in the agroecosystem affect the pattern of vector abundance and vary between years, especially for overwintering adults^48,49^.

The non-detection of MRDV in any of the analysed weeds confirms the reduced number of alternative hosts of this virus as well as their reduced susceptibility^14,16,18,20^. Most of the grasses found to be infected in these studies were summer or late-spring grasses, as sampling was performed in very late spring or summer, while our sampling was conducted when summer grasses were rather scarce, and sampling was focused on the most abundant grasses. On the other hand, the non-detection of MRDV may be due to the number of samples analysed; therefore, future analyses would have to be carried out with a more sensitive method. In this context, the results obtained using a next-generation sequencing (NGS) approach confirmed that *Avena* spp. is a host of MRDV (unpublished data).

The results obtained in this study show the effect of surrounding crops and their management on the epidemiology of MRDD. We report that higher incidences were observed in the late planting period, and the effects of the main variables implicated in the MRDV incidence varied with planting period. In addition, the strong influence of maize planting period on MRDV incidence, vector abundance, grass plant cover at the edges and the proportion of winter cereals/fallow in the landscape are the main factors involved in the epidemiology of MRDV (Fig. 3). The contribution of the factors that determine virus incidence strongly depends on the crop planting period. In the early planting period, the presence of edges is the main factor to consider, while in the late planting period, the increase in vector abundance increases the risk of infection. In addition, our results show that *L. striatellus* numbers are related to MRDV in the late planting period, which has not been previously reported in our area and is in contrast to the results of Wang et al*.*^26^ but consistent with the results of Conti^38^. The patterns of the movement and abundance of the species in agricultural landscapes are highly complex (temporal and spatial), and this complexity hinders the interpretation and comparison of these parameters among studies^50^; thus, this aspect should be studied more thoroughly in future research.

These results contribute to our knowledge of influence crop management practices on MRDV incidence and could be considered when selecting planting periods to minimize the virus incidence in maize crop areas. Finally, a number of recommendations could be issued from this study to minimize the risk of infection by MRDV, which is responsible for substantial losses in maize production in our area:

1. Late maize planting periods should be avoided as much as possible to minimize the risk of infection by MRDV; from this perspective, March and April could be suitable planting months.
2. Simultaneous planting of maize fields in the vicinity of winter cereals should be avoided as much as possible, especially in cereal harvesting periods.
3. The application of herbicides at edges could be optimized to minimize the grass cover in both the planting period and the first stage of crop development. Grass species act as sources of the viral inoculum and as breeding and feeding vectors. However, it should be considered that edges can also be a reservoir of natural enemies that colonize maize fields and prevent outbreaks of insect pests.

## Methods

### Study area

**Figure 4:**
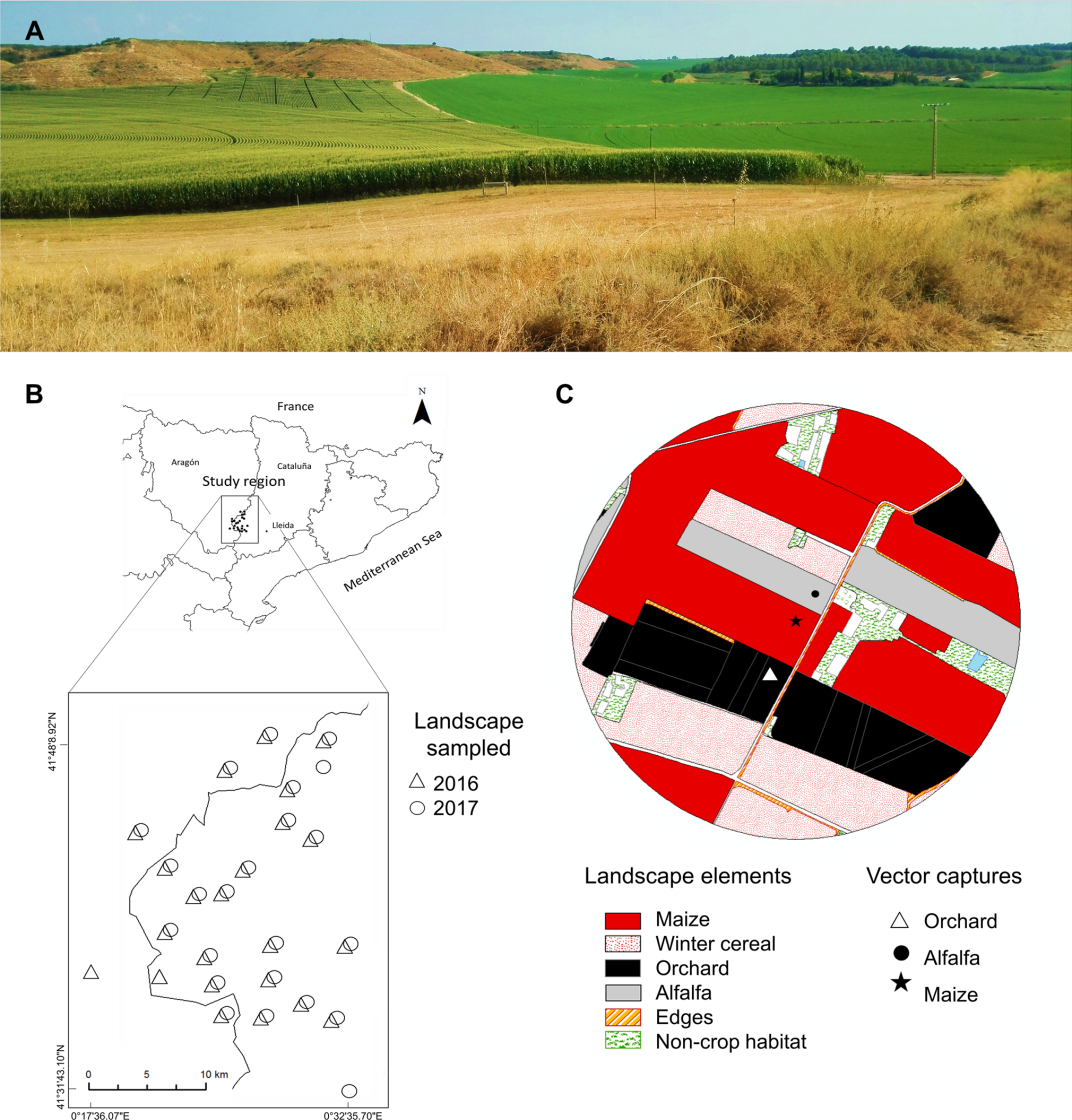
Study region in the Ebro Basin in northeastern Spain (**A**). Landscape sampled in 2016 and 2017 (**B**). The star, the circle and the triangle indicate the middles of the sticky yellow traps in the sampled fields used to collect MRDV vectors (**C**).

The study was carried out during 2016 and 2017 in the Ebro Basin in northeastern Spain (41° 48′ 12.20″ N, 0° 32′ 45.77″ E; 120–346 m altitude; 200–400 mm rainfall; Tmin: 8°–24 °C and Tmax: 18°–38 °C). The agroecosystem has been traditionally dominated by alfalfa in rotation with winter (mainly wheat and barley, from December to June) and summer (mainly maize, from March to November) cereals. Recently, commercial demand has led to an increase in stone fruit orchard surfaces in the area, leading to a more intensive agricultural landscape that is interspersed with scattered patches of non-crop habitats (non-productive areas, long fallows, semi-natural habitats and forests repopulated by *Pinus halepensis* (Mill)) (Fig. 4A). Common pest management efforts in these crops in our area include the following: (1) cereals: pre- and post-emergence herbicide applications, seed treatment with both insecticides and fungicides; (2) alfalfa: 5/6 cuttings during the productive period (March–October), crop is in the field for 4–5 years^51^; (3) orchards: management includes an average of 7–14 chemical sprays (insecticides, fungicides and bioregulators), herbaceous cover mowing (approximately once per month), herbicide application (mainly glyphosate), tree fertilization^52^.

### Variables sampled in maize fields

Forty-six fields were selected in 2016 and 2017 in areas with different gradients of cereal proportions in the surrounding landscapes. A few fields changed in the two years due to rotation. The size of the fields varied between 0.9 and 26.13 ha. The sampled maize fields were separated by at least 2 km so that the study spanned an agricultural landscape of 700 km^2^ (Fig. 4B). The maize field variables considered by the analysis were maize field surface area (ha) and planting period (number of weeks in the year).

### Maize survey

A random survey for MRDV incidence was conducted at maize anthesis following the scheme described by Achon and Sobrepere^15^ in July. In each field, we randomly collected the three upper leaves of approximately 30 maize plants following a W-shaped pattern. The distance between plants varied according to the maize field size. In each of the fields, each sample was placed separately in a plastic bag and stored at − 80 °C until virus identification.

### Edge surveys for weed and grass collection

Floristic surveys were conducted at the edges of the 46 maize fields surrounded by crop or non-crop areas during May–June in the 2 years. To determine the abundance and composition of plant species at the edges, especially grass species, we carried out surveys in edge areas when the maize was in the early growth stage. For each sampling point, the cover abundance of weed species was recorded using the Braun-Blanquet scale^53^ in three rectangular plots (2 × 5 m^2^) along the edges. The number of edges surveyed in each landscape was between 2 and 6 but depended on the numbers of different crop and non-crop habitats close to the sampled maize field. For instance, in very diverse landscapes, we sampled 6 edges: maize-orchard, maize-alfalfa, orchard-alfalfa, maize-maize, orchard ground cover, and non-crop habitats. Then, the cover abundance values were transformed into the mean value of the percent cover of each field, and the Shannon index (H′) was calculated (Eq. (1)):

1 $${\text{H}} = - \sum\limits_{i = 1}^{46} {\pi_{i} \times ln\,\pi_{i} }$$

where ‘π’ is the proportional abundance of a species, and ‘*i*’ is the number of observations. H′ and the grass cover proportion were the explanatory field variables in the models.

In addition, to provide information about the cover plants and diversity groups of the edges, we used floristic surveys to transform the cover abundance of species to the mean value of the percent cover according to six types of edges sampled to calculate the Shannon index. These variables were only descriptive and were not included in the analysis.

Furthermore, to detect putative alternative hosts of the virus, we collected samples in the surveyed edge plots mentioned above according to the following criteria: (1) two samples of the most abundant grass, (2) one sample of the second most abundant grass, and (3) two samples of the least abundant grass species. In each field, each sample was placed in a separate plastic bag, identified at the species level, and examined for virus-like symptoms or no symptoms. All samples were stored at -80 °C until virus identification.

### Virus detection

Maize and grass samples were examined for MRDV symptoms; additionally, virus infection of symptomatic maize samples was verified by selective isolation of genomic dsRNA segments of MRDV using the modified mini-prep method of DePaulo and Powell^54^. Briefly, dsRNA was isolated from 40 mg of fresh tissue by the sodium dodecyl sulphate (SDS)/KOAc procedure, fractionated by chromatography on non-ionic cellulose (CF-11), eluted in 30 μl of RNAase-free water and separated on 0.8% agarose gels to observe the presence of the MRDV genomic segments. This method was also used to detect virus infection in grass samples with or without symptoms.

### *Laodelphax striatellus* data collection

To determine the contribution of *L. striatellus* to the different crops in the landscape, samplings were performed in maize fields, alfalfa fields and orchards (Fig. 4C). *L. striatellus* was captured using yellow sticky traps (30 × 25 cm, Serbios, Badia Polesine, Italy). Five samplings were performed monthly using 3 traps per field (3–9 traps per locality), and traps remained active for 7 days during the maize growing season. A total of 1,812 traps were placed in the fields over the two years. In maize fields, the traps were placed on a stake at canopy height (until V12) or at ear level (from V15 onwards) depending on the growth stage, and they were arranged in a transect perpendicular to the edge, with a separation distance of 15 m (the first one was placed 15 m from the edge)^55^. In alfalfa fields, traps were placed on a stake at the canopy level, with a height of 1 m, in a transect perpendicular to the edge, and traps were separated from each other by 12 m, with the first trap located 12 m from the edge^56^. In orchards, traps were placed on a stake at a height of 2 m within tree lines and were separated from each other by 30 m, starting 30 m from the edge. Once collected, the samples were kept at 4 °C until processing. The number of *L. striatellus* individuals caught on each trap was counted under binoculars and identified at the species level using the key of Holzinger et al.^57^.

### Variables at the landscape level

The maize fields were selected based on the proportion of cereals in the landscape using aerial photography in a circular buffer of 500 m surrounding the maize fields (Fig. 4C). The landscape composition was characterized by the proportions of the different landscape elements embedded in the circular buffer surrounding the maize fields. To incorporate the seasonal variation in the proportion of cereals in early and late spring in the landscape, the composition was measured in the two periods coinciding with the early and late maize planting periods. The landscape composition was described each year by direct field observations, by an orthophoto of Plan Nacional de Ortografan Aérea (PNOA), and by geographical information maps of the Instituto Geográfico Nacional of Spain. Then, we quantified the proportions of the landscape elements using ArcGIS software 10.3.1^58^. Next, the 34 landscape elements initially identified in the study were grouped into seven categories: orchards, maize, winter cereals, winter cereal/fallow, alfalfa, non-crop habitats and edges (Fig. 4C).

### Data analysis

Data on virus incidence in maize fields were not normally distributed and were transformed by (log x + 1). To identify the influence of the planting month on virus incidence, we analysed the number of plants infected by MRDV in each field with a two-way ANOVA, including the month and year as factors, while the number of maize samples per field used to analyse virus presence and the area of maize fields were used as covariates. The month × year interaction was not significant and was removed from the analysis.

We used multi-model inference (‘MuMIn’ package^59^, a procedure that fits models using all possible combinations of predictors and then weights them by the Akaike information criterion (AIC) (dredge function). This method allows the data-based selection of a “best” model and a ranking and weighting of the remaining models in a predefined set. This procedure entailed generating AIC values and Akaike weights for each candidate model. Model averaging was performed with a setting of ΔAICc < 2^60^. The selection of a best approximating model represents the inference from the data and tells us what “effects” (represented by parameters) can be supported by the data. First, we used Moran’s I statistic^61^ to determine whether there was spatial autocorrelation (measure of the correlation of a variable with itself through space) regarding the incidence of MRDV and *L. striatellus* abundance. The results indicated that there was no significant spatial autocorrelation (MRDV Moran’s I = 0.11, *p* = 0.14; *L. striatellus* Moran’s I = -0.07, *p* = 0.6). Moreover, the landscape and field metrics for each model were standardized (mean centred and scaled) using the ‘caret’ package^62^. Then, the relationships between the incidence of MRDV and the field and landscape variables were analysed using generalized linear models (GLMs) with the ‘lme4’ package^63^ (for binomial distribution) by each planting period (early: March and April; late: May and June). We used the percentage of viral incidence in each field including the weight of the variable (number of maize samples per field) to analyse the effects. Models of MRDV included the following fixed factors: planting period (number of weeks in the year); area of maize field (ha); diversity of edges (Shannon index); proportions of alfalfa, maize, winter cereal/fallow, orchard, edges, and non-crop habitats; and year and sum of *L. striatellus.* Then, model residuals were graphically inspected with qqplot and histogram graphics to ensure there was no violation of the normality and homoscedasticity assumptions^64^. Finally, in the MRDV models, the relative importance of each predictor variable was plotted to check the weight of the variables included in the best models.

Data on *L. striatellus* abundance captured in the landscape showed no homogeneity of variances, and we used a Kruskal–Wallis test for analysis. We compared the vector abundance among months in 2016 (March, May, June, July, August and September) and 2017 (May, June, August, September and October). Further differences were analysed using the Dunn test.

Finally, the models relating MRDV incidence to *L. striatellus* abundance captured by crop and by month (May, June and July) were analysed using a generalized linear mixed model (GLMM) for binomial distribution, including the year as a random factor with the ‘lme4’ package. All analyses were performed using R software^65^.

## Supplementary Information

Supplementary Information.
