# Supplementary material for: Planting period is the main factor for controlling maize rough dwarf disease
Source: Sci Rep. 2021 Jan 13;11:977. doi: 10.1038/s41598-020-79994-5 (PMC7806615; doi:10.1038/s41598-020-79994-5)
Supplement: Supplementary file 1 — Supplementary Information. [file 41598_2020_79994_MOESM1_ESM.docx]

*Supplementary Information*

Planting period is the main factor for controlling maize rough dwarf disease

Gemma Clemente-Orta, Ramon Albajes, Iván Batuecas and Maria Angeles Achon

This document contains Table S1.

Table S1. MRDV incidences registered in maize fields during 2 samplings years.

| Year | X coordenate | Y coordenate | Maize samples | MRDV incidence |
| --- | --- | --- | --- | --- |
| 2016 | 41,72556389 | 0,502105556 | 31 | 0 |
| 2016 | 41,58413333 | 0,528597222 | 30 | 0,066666667 |
| 2016 | 41,59640556 | 0,498297222 | 29 | 0,034482759 |
| 2016 | 41,61572778 | 0,464677778 | 30 | 0 |
| 2016 | 41,77586944 | 0,416872222 | 30 | 0 |
| 2016 | 41,80128333 | 0,451394444 | 30 | 0,2 |
| 2016 | 41,61586667 | 0,29285 | 33 | 0 |
| 2016 | 41,76372778 | 0,480488889 | 30 | 0 |
| 2016 | 41,64130278 | 0,468463889 | 30 | 0,066666667 |
| 2016 | 41,62983611 | 0,402233333 | 30 | 0,1 |
| 2016 | 41,68131389 | 0,4143 | 32 | 0 |
| 2016 | 41,64766667 | 0,365869444 | 31 | 0 |
| 2016 | 41,64247778 | 0,540616667 | 30 | 0,2 |
| 2016 | 41,73740556 | 0,475555556 | 30 | 0 |
| 2016 | 41,58572222 | 0,459930556 | 30 | 0,466666667 |
| 2016 | 41,60918889 | 0,41255 | 30 | 0,266666667 |
| 2016 | 41,61281111 | 0,356002778 | 31 | 0,483870968 |
| 2016 | 41,6758 | 0,389116667 | 29 | 0,137931034 |
| 2016 | 41,70059167 | 0,363402778 | 30 | 0,166666667 |
| 2016 | 41,58521111 | 0,425672222 | 31 | 0,451612903 |
| 2016 | 41,80146944 | 0,509402778 | 28 | 0 |
| 2016 | 41,70012778 | 0,438494444 | 30 | 0 |
| 2016 | 41,72471389 | 0,331738889 | 31 | 0,580645161 |
| 2017 | 41,72556389 | 0,502105556 | 30 | 0 |
| 2017 | 41,58413333 | 0,528597222 | 29 | 0,206896552 |
| 2017 | 41,59640556 | 0,498297222 | 30 | 0,033333333 |
| 2017 | 41,61572778 | 0,464677778 | 30 | 0,033333333 |
| 2017 | 41,77586944 | 0,416872222 | 30 | 0 |
| 2017 | 41,80341667 | 0,450741667 | 30 | 0,1 |
| 2017 | 41,77796667 | 0,509972222 | 30 | 0,1 |
| 2017 | 41,76372778 | 0,480488889 | 30 | 0 |
| 2017 | 41,64130278 | 0,468463889 | 30 | 0,166666667 |
| 2017 | 41,52848611 | 0,544041667 | 30 | 0,133333333 |
| 2017 | 41,62983611 | 0,402233333 | 30 | 0 |
| 2017 | 41,68131389 | 0,4143 | 30 | 0 |
| 2017 | 41,64766667 | 0,365869444 | 30 | 0,233333333 |
| 2017 | 41,64247778 | 0,540616667 | 30 | 0,3 |
| 2017 | 41,73740556 | 0,475555556 | 30 | 0 |
| 2017 | 41,58515833 | 0,458816667 | 30 | 0,366666667 |
| 2017 | 41,60918889 | 0,41255 | 30 | 0 |
| 2017 | 41,67521111 | 0,389169444 | 30 | 0,1 |
| 2017 | 41,70169167 | 0,365136111 | 30 | 0,033333333 |
| 2017 | 41,58553611 | 0,425955556 | 30 | 0,166666667 |
| 2017 | 41,79966944 | 0,509863889 | 29 | 0,068965517 |
| 2017 | 41,70012778 | 0,438494444 | 30 | 0 |
| 2017 | 41,72471389 | 0,331738889 | 30 | 0,133333333 |
